# Effect of Polishing Systems on the Surface Roughness and Color Stability of Aged and Stained Bulk-Fill Resin Composites

**DOI:** 10.3390/ma17143576

**Published:** 2024-07-19

**Authors:** Ayşem Aktu, Nuran Ulusoy

**Affiliations:** Faculty of Dentistry, Department of Restorative Dentistry, Near East University, 99138 Nicosia, Türkiye; nuran.ulusoy@neu.edu.tr

**Keywords:** single-shade bulk-fill, aging, SEM, surface roughness, color stability, polishing

## Abstract

The aim of this study is to investigate how two different polishing techniques affect the color stability, surface roughness, and changes in surface morphology of aged and coffee-stained bulk-fill resin composites. A total of 112 disc-shaped samples were prepared using Omnichroma Flow Bulk (OB), Charisma Bulk Flow One (CB), Estelite Bulk Fill Flow (EB), and Estelite Sigma Quick (control). Samples were divided into two subgroups (n = 16) and aged using thermocycling. A profilometer was used to measure the surface roughness (R_a_) and a spectrophotometer was used for color stability (ΔE_00_). The EB group had the highest Ra values both before aging (disc: 0.23 ± 0.05, twist: 0.42 ± 0.05) and after aging (discs: 0.28 ± 0.04, twist: 0.46 ± 0.05). The OB group had the highest ΔE_00_ values before and after aging (discs: 3.06 ± 0.54, twist 3.05 ± 0.41) and the highest after coffee-staining (discs: 3.75 ± 0.70, twist: 3.91 ± 0.57). Re-polishing reduced the ΔE_00_ values in all materials but did not restore all to clinically acceptable levels. According to the results of this study, it can be concluded that the surface roughness and color stability of resin composites are notably influenced by the polishing technique, aging process, and coffee staining. Specimens that were polished using the multi-stage Super-Snap discs consistently exhibited smoother surfaces across all bulk-fill resin composites compared to those polished with the two-stage Diacomp plus Twist.

## 1. Introduction

Resin composites are frequently used in dentistry to meet the increasing aesthetic expectations of patients in terms of their optical and mechanical properties [1]. In order to improve the wear resistance and polishability of earlier macro- and micro-filled resin composites, changes in the ingredients have been made. An increased filler load and decreased filler size resulted in the creation of microhybrid and nanohybrid resin composites [2]. However, this increase in filler load also led to greater rigidity and curing stress, necessitating the use of appropriate layering techniques. It is recommended to utilize a stratified technique during the application of traditional resin composites. Applying the composite resin in 2 mm increments can extend the time required for application, especially in deeper cavities [3,4]. Nowadays, bulk-fill resin composites with an increased filler content are available, eliminating the necessity for additional composite layers. The first bulk-fill resin composites were designed as base materials to be topped with a conventional resin composite for the occlusal layer. This initial category of bulk-fill composites is known as “base” or “low viscosity” composites. More recently, bulk-fill composites with a higher filler load have been introduced as one-step materials that do not require an occlusal capping layer. This newer category is classified as “full-body” or “high viscosity” composites, which are to be applied to deep cavities [2,5].

Compared to conventional composite resins, bulk-fill composite resins are frequently used today due to advantages such as reducing time-consuming techniques in deep cavities, being able to fill cavities up to 4–5 mm in a single increment, and reducing polymerization shrinkage. Recently, single-shade bulk-fill composite resins, which have physical properties similar to traditional bulk-fill composites, have been produced by various companies. This not only simplifies the workflow but also eliminates the need for shade selection. Their usage may increase due to their ability to be placed up to 3.5–4 mm in a single increment and their aesthetic properties.

In our study, Estelite Bulk Fill Flow (Tokuyama, Tokyo, Japan), which is a methacrylate-based low viscosity bulk-fill resin composite, and two urethanedimethacrylate-based bulk-fill resin composites, Omnichroma Flow Bulk (Tokuyama, Tokyo, Japan) and Charisma Bulk Flow One (Kulzer, Leipziger, Germany), were used. Omnichroma Flow Bulk features a low filler content and low viscosity, making it suitable for application in 3.5 mm increments [6]. Similarly, Kulzer introduced Charisma Bulk Flow One, an advanced flowable composite for dental restorations [7]. This material stands out as the first flowable, single-shade bulk-filling composite that does not require an additional capping layer, which significantly simplifies the restorative procedure. Additionally, Tokuyama Dental’s Estelite Bulk Fill Flow can be applied in layers up to 4 mm thick without needing a closure layer and is available in five shades [8].

For a successful aesthetic restoration, beyond the application technique, the material chosen must possess specific properties. These include the composition of particles and resin matrix, surface smoothness, and color matching, all of which should mimic the natural tooth structure closely [1,9]. The shrinkage stress of bulk-fill composites is low; however, the composites have a high degree of polymerization due to the increase in translucency and the presence of polymerization modulators [10]. In addition to the mentioned properties, bulk-fill resin composites have been reported to have disadvantages such as surface degradation, marginal fractures, discoloration, polymerization shrinkage, and plaque accumulation that gives way to surface roughness [11]. Surface roughness is an important factor that affects the color change of resin composites.

The change in color is quantified through Delta E (ΔE*) and combines the differences in the L*, a*, and b* parameters into a single combined measure [12]. Various ΔE* thresholds have been proposed to define the threshold for what qualifies as “clinically acceptable” discoloration. The CIE Lab* scale, devised by the International Commission on Illumination (CIE, Commission Internationale d’Eclairage), delineates the color attributes of an object utilizing these three parameters. While the CIELAB formula is frequently employed for aligning the color of restorative materials, the CIEDE2000 formula is presently the preferred approach for assessing ΔE_00_ [13].

Discoloration in composite restorations over time can be of internal origin or external origin due to plaque accumulation and diet [14,15]. Various factors can lead to the extrinsic discoloration of resin composites, such as the use of mouthrinses and the consumption of acidic or staining foods and drinks in the oral environment. Previous research indicates that beverages like coffee, tea, red wine, and cola can induce discoloration on light-curing resin composite restorations [5,16]. Polishability is a crucial property of resin composites as surface characteristics like roughness significantly influence the clinical outcomes of restorations. Inadequately finished and polished surfaces are more susceptible to wear and plaque accumulation, increasing the risk of staining, secondary caries, and gingival irritation, which can compromise clinical success. Polishing dental restorations is crucial to slow down the discoloration and aging processes of composite resins. In order to investigate the changes due to aging, different mechanisms have been proposed. Artificial aging can be used to analyze both short-term and long-term effects; the artificial aging process causes material degradation, which may result in alterations to the mechanical and optical properties of composite restorations [17].

Resin composite restoration achieves its optimal condition when the resin undergoes polymerization while placed against a suitable mylar strip. Without the use of a mylar strip, the polymerization process of the outer layer is impeded, resulting in a surface layer that is rich in organic binder, causing a tackier, softer texture. As this texture is not sustainable, additional contouring and finishing becomes necessary. Finishing involves shaping the restoration to achieve the desired anatomy, whereas polishing entails smoothing the surface to eliminate roughness and scratches caused by finishing tools. Additionally, it is well-known that restorations with smooth surfaces are more comfortable, aesthetically pleasing, and better accepted by patients [18].

In the previous studies, it has been reported that surface roughness values of less than 0.2 μm are considered ideal for intraoral restorations and a restoration that is rougher than 0.2 μm makes bacterial retention easier [19,20]. Minor changes in surface roughness, typically around 0.3 µm, can be easily detected by the tip of the tongue, resulting in a noticeable roughness [21,22]. Therefore, finishing and polishing procedures are crucial not only for creating a standardized aesthetic, but also for the longevity of the composite restoration. The effects of polishing systems on the surface roughness of resin composites do not result from a single factor and several factors impact the surface properties of restorative materials. Hence, it is necessary to employ diverse polishing systems, procedures, and materials. With a plethora of polishing systems available, it is crucial to assess them to determine which delivers the optimal polishing effect on specific resin composites.

Achieving the desired glossy surface during finishing and polishing procedures necessitates the utilization of a sequence of instruments featuring progressively finer abrasives. Conventionally, highly flexible multi-step polyurethane-based discs coated with aluminum oxide were frequently employed for polishing resin composite restorations. More recently, diamond polishers and silicone synthetic rubbers have emerged as alternatives. These newer tools provide hybrid composites with surface shine and also reduce the clinical time required to finish the restoration. However, inconsistencies in the scientific literature regarding these matters, along with modern composite materials and polishing systems, emphasize the necessity for additional research on this subject.

Therefore, the objective of this study was to assess the impact of two different finishing and polishing systems on the surface roughness and color stability of single-shade and multi-shade bulk-fill resin composites both before and after aging.

The effect of re-polishing on the color stability of single-shade and multi-shade bulk-fill composite resins colored with coffee was also investigated. This study was conducted using the following alternative hypotheses:

**H_A_1:**  *Various polishing techniques impact the surface texture of bulk-fill composite resins.*

**H_A_2:**  *Various polishing techniques impact the color stability of bulk-fill composite resins.*

**H_A_3:**  *Bulk-fill resin composites with single shades and multi shades polished with different polishing systems exhibit contrasting surface roughness characteristics.*

**H_A_4:**  *Single-shade and multi-shade bulk-fill composite resins polished with different polishing systems differ from each other in terms of color stability.*

**H_A_5:**  *Re-polishing improves the color of coffee-stained resin composites.*

## 2. Materials and Methods

### 2.1. Preparation of Samples

**Figure 1:**
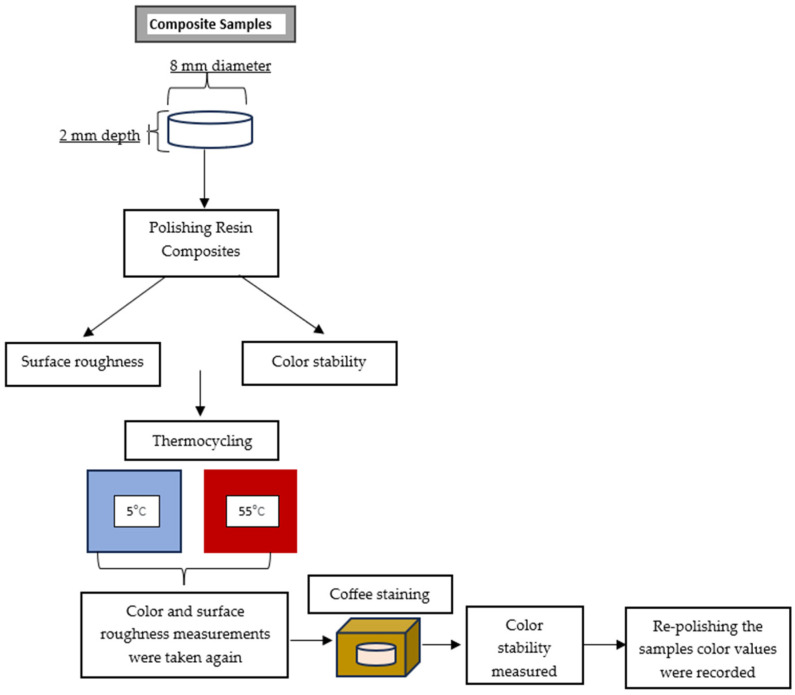
Schematic workflow of the study.

Two single-shade bulk-fill resin composites, Omnichroma Flow Bulk (Tokuyama, Tokyo, Japan) and Charisma Bulk Flow One (Kulzer, Germany), and the A1 shade of Estelite Bulk Fill Flow (Tokuyama, Tokyo, Japan) were used in the study. The A1 shade of Estelite Sigma Quick (Tokuyama, Tokyo, Japan) was used as the control group (Table 1). The schematic workflow of the study is given in Figure 1.

Teflon mold was used to prepare the samples. Resin composites were placed in the round holes (8 mm × 2 mm) drilled in the Teflon plate, covered on each side with a Mylar strip, pressed with a smooth glass plate to remove excess, and polymerized with a 1000 mW/cm^2^ intensity LED light unit (Woodpecker DTE Curing Light LED (1 s cure)/China) for 20 s. A curing radiometer (L.E.D.Curing radiometer, Demetron, SDS/Kerr, Orange, CA, USA) was used to check the intensity of the LED light unit. A total of 112 disc-shaped specimens were prepared and randomly divided into three groups (n:32), which were later divided into two subgroups (n:16). The control group consisted of 16 specimens. All the test groups were either polished using the multi-step disc system (Super-Snap Rainbow Kit, Shofu, Kyoto, Japan) or two-step twist system (Diacomp plus Twist, EVE Technik, Pforzheim, Germany).

### 2.2. Polishing of Resin Composites

**Figure 2:**
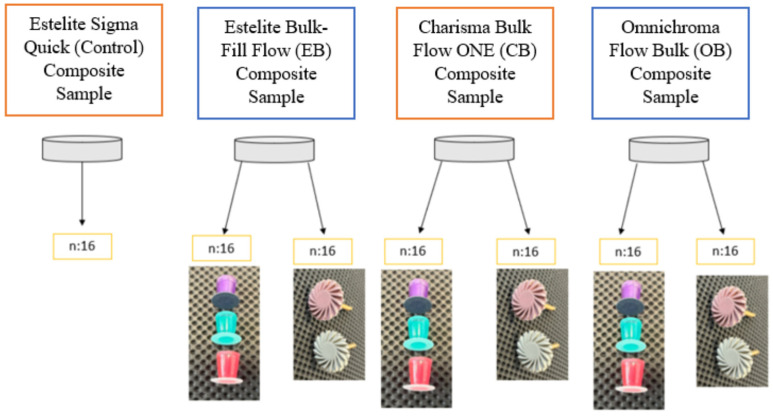
Polishing application procedure.

The control group containing 16 specimens was not subjected to any polishing procedure. The other test specimens were either polished using the disc or twist systems (Figure 2). Super-Snap Rainbow Discs (Shofu, Kyoto, Japan) with silicon carbide and aluminum content were used. The discs were applied as a medium, fine and super fine respectively. Diacomp plus Twist (EVE Technik, Pforzheim, Germany) with diamond content was used for medium and fine grain. Polishing processes were applied underwater. All samples were used sequentially for 15 s under water cooling at a constant speed of 10,000 rpm with light pressure.

### 2.3. Surface Roughness

Following 24 h of immersion in distilled water, the surface roughness (Ra) was measured using a profilometer (Perthometer M2, Mahr GmbH, Gottingen, Germany).

The profilometer had a tip diameter of 2.4 mm and an accuracy of 0.5 mm/s, with a tracing length set to 5.5 mm. For each specimen, 3 measurements were made, and the arithmetic mean values were used for statistical analysis. The samples were then exposed to artificial aging (5 and 55 °C, 5000 cycles, 30 s each) using a thermocycler (SD Mechatronik Thermocycler, SD Mechatronik GMBH, Westerham, Germany). After the aging process, the surface roughness values (Ra) of the samples were measured and recorded again.

### 2.4. Staining Protocol

The specimens, grouped according to polishing methods, were then submerged in a coffee solution (one teaspoon of coffee in 180 mL of freshly boiled water, Nescafe Classic, France). The control group was kept in distilled water. Studies have reported that a coffee consumer drinks 3.2 cups of coffee daily and assigns 15 min for the consumption of one cup of coffee. Based on this knowledge, a storage period of 24 h was reported to simulate the consumption of the beverage for 1 month [16]. The specimens were immersed in their respective coffee solutions at 37 °C for six days, simulating approximately six months of coffee exposure. The coffee solution was changed every day. After the staining period was completed, the samples were rinsed for 10 s, air-dried, and then color parameters were recorded. They were then re-polished using Super-Snap Rainbow Discs or Diacomp plus Twist and the color parameters were recorded once again.

### 2.5. Color Stability

Initial color measurements for all samples were captured immediately following the completion of sample preparation. The color characteristics of each specimen were assessed at four distinct time intervals: immediately after preparation, following aging, subsequent to staining, and ultimately after re-polishing. The samples were measured using a spectrophotometer (Vita Easyshade Compact, Model #DEASYCHP, Vita Zahnfabrik, Bad Sackingen, Germany) under D65 lighting conditions in a windowless dental clinic room at 25 °C [23]. The color difference of each specimen was determined using the CIEDE2000 formula (ΔE_00_). The CIEDE2000 color difference (ΔE_00_) was computed utilizing a spreadsheet application in Excel, employing the CIEDE2000 color difference formula outlined by Lou, Cui, and Rigg [13]. The parametric factors of the formula have been established as 2.1.1 [24]. According to this formula, the acceptable detection range for the color parameter ΔE_00_ is between 0.8 and 1.8 at the 50–50% level [25].

### 2.6. SEM Evaluation

Samples were fixed to SEM plates (ZEISS EVO MA 40, ZEISS, Oberkochen, Germany) with gold conductive tape and gold coated in a vacuum sputter coater (Emitech K550X Sputter Coater, Ashford, Kent, UK). This instrument is equipped with a 60 mm diameter, 0.1 mm thick, gold, quick-change target. SEM photomicrographs of the polished surfaces of the samples were taken under 500× and 2000× magnification.

### 2.7. Statistical Analysis

The sample size for the study was established at a 95% confidence level using the “G.Power-3.1.9.2” software. As a result of the analysis, when α = 0.05, a standardized effect size was obtained as 0.5518 from a similar study (Paolone, G., et al., (2020); 54.9 ± 15.4, 45.0 ± 11.3, 38.4 ± 11.6 and 52.6 ± 9.0) [2]. The minimum sample size for each group was calculated as 16 with a theoretical power of 0.95. The analyses were conducted using the IBM SPSS 25 program. The normality assumption of the statistical analysis was verified using the Shapiro–Wilk test, while homogeneity of variance was assessed through Levene’s test, and the sphericity assumption was evaluated via Mauchly’s W test. The Two-Way Repeated Measures ANOVA test was performed to examine the difference between the averages of the three dependent groups in which the normality assumption was met with the interaction effect.

## 3. Results

### 3.1. Surface Roughness

**Figure 3:**
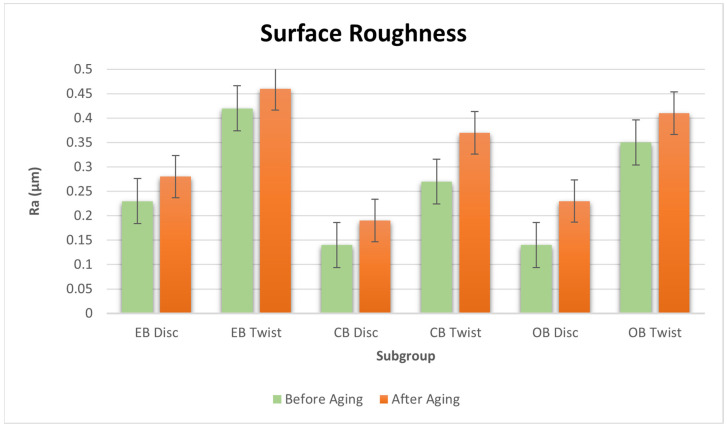
Mean R_a_ values (μm) of resin composites before and after aging according to the tested finishing and polishing procedure.

The distribution of Ra measurements according to measurement time and groups is shown in Table 2 and Figure 3. The analyses conducted for the disc and twist groups before and after aging revealed statistically significant differences in the Ra measurements among the different study groups (*p* < 0.05). Based on the before-aging assessments of the disc study groups, it was noted that Ra measurements for the CB (0.14 ± 0.05), OB (0.14 ± 0.05) and EB (0.23 ± 0.05) groups were higher compared to those of the control group (0.03 ± 0.00). Similarly, for the twist study groups, the Ra measurements for the CB (0.27 ± 0.08), OB (0.35 ± 0.05), and EB (0.42 ± 0.05) groups were also higher than those of the control group (0.03 ± 0.00). In the Ra measurements between the CB and EB groups, the EB group exhibited the higher value. According to the tests conducted for the disc study groups after aging, the Ra measurements of the OB (0.23 ± 0.05) and EB (0.28 ± 0.04) groups were higher than that of the control group (0.12 ± 0.04). Additionally, the Ra measurement of the EB group (0.28 ± 0.04) was higher than that of the CB group (0.19 ± 0.05).

According to the tests conducted for the twist study groups, the Ra measurements of the CB (0.37 ± 0.06), OB (0.41 ± 0.08), and EB (0.46 ± 0.05) groups were higher than the Ra measurement of the control group (0.12 ± 0.04). The Ra measurements of the twist group before and after aging were higher than the Ra measurements of the disc group. Statistically significant differences were obtained between Ra measurements according to the time in the disc and twist groups, OB, CB, EB, and control groups (*p* < 0.05). In all groups, the Ra measurements after aging were higher than the pre-aging Ra measurements.

### 3.2. Color Stability

**Figure 4:**
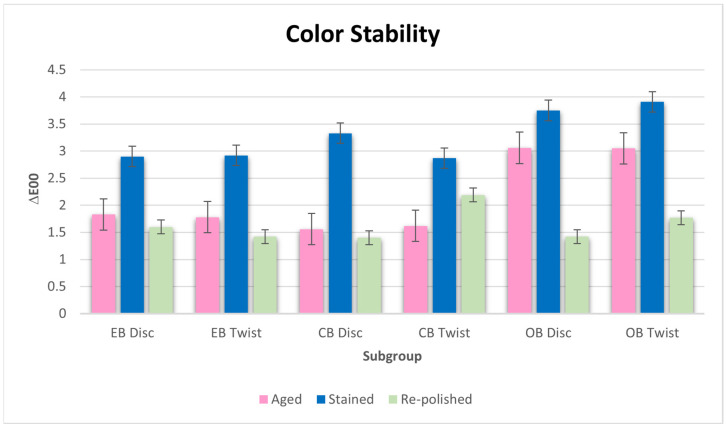
Mean (ΔE_00_) values of resin composites before and after aging, after coffee staining, and after re-polishing according to the tested finishing and polishing procedure.

The ΔE_00_ values of all samples were taken after aging, staining, and re-polishing (Table 3, Figure 4). Statistically significant differences (*p* < 0.05) were observed in the disc and twist groups after aging, coloring with coffee, and re-polishing. In both groups, despite a significant color change, the CB group maintained a value within the clinically acceptable range. According to the tests performed for the ΔE_00_ disc study groups after aging, the coloration measurements of the OB (3.06 ± 0.54), EB (1.83 ± 0.42), and CB (1.56 ± 0.32) groups were higher than the coloration measurement of the control group (1.26 ± 0.25). According to the tests performed for the twist study groups after aging, the coloration measurements of the OB (3.05 ± 0.41), EB (1.78 ± 0.39) and CB (1.62 ± 0.45) groups were higher than the coloration measurement of the control group (1.26 ± 0.25). The OB group exhibited the highest ΔE_00_ value among the after aging measurements, with values from both disc and twist polishing being in the clinically unacceptable range. None of the tested samples showed a clinically acceptable value after coloration with coffee. In all samples, the coloration measurement of the twist group was higher than the coloration measurement of the disc group. The OB group showed the highest ΔE_00_ value after both disc and twist-polished samples after being immersed in coffee. Following re-polishing, only the CB group exhibited a statistically significant disparity in coloration measurements when compared to the clinically acceptable thresholds of the disc and twist groups.

### 3.3. SEM Images of Surface Topography

**Figure 5:**
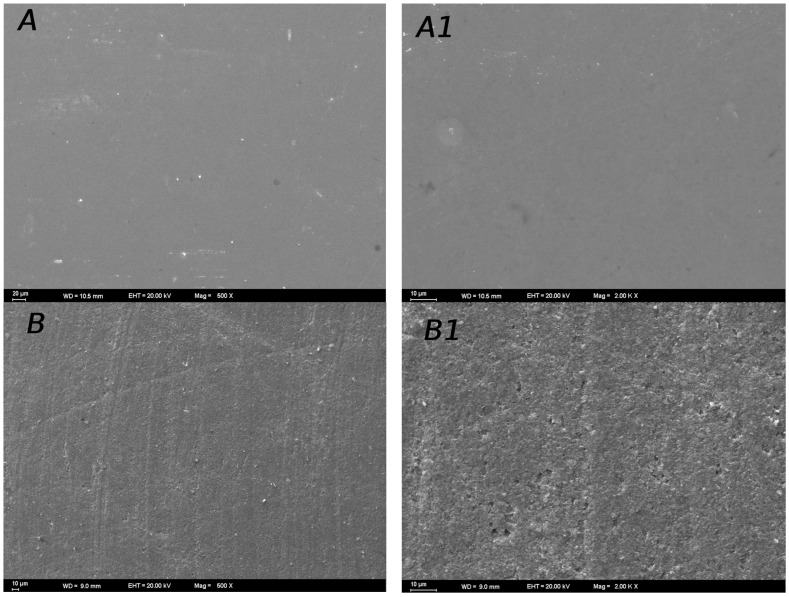

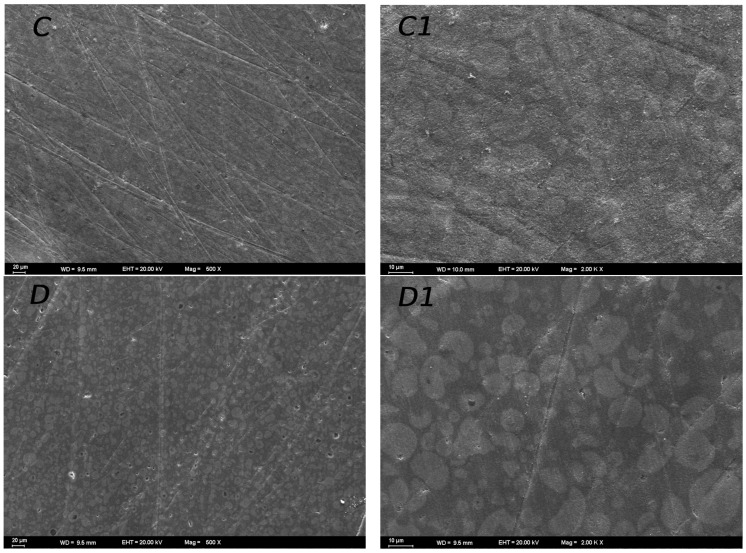
SEM images of the samples polished with discs. (**A**) Estelite Sigma Quick 500X (Mylar strip), (**A1**) Estelite Sigma Quick 2000X (Mylar strip), (**B**) Charisma Bulk Flow One500×, (**B1**) Charisma Bulk Flow One 2000×, (**C**) Omnichroma Flow Bulk 500×, (**C1**) Omnichroma Flow Bulk 2000×, (**D**) Estelite Bulk Flow 500×, and (**D1**): Estelite Bulk Flow 2000×.

**Figure 6:**
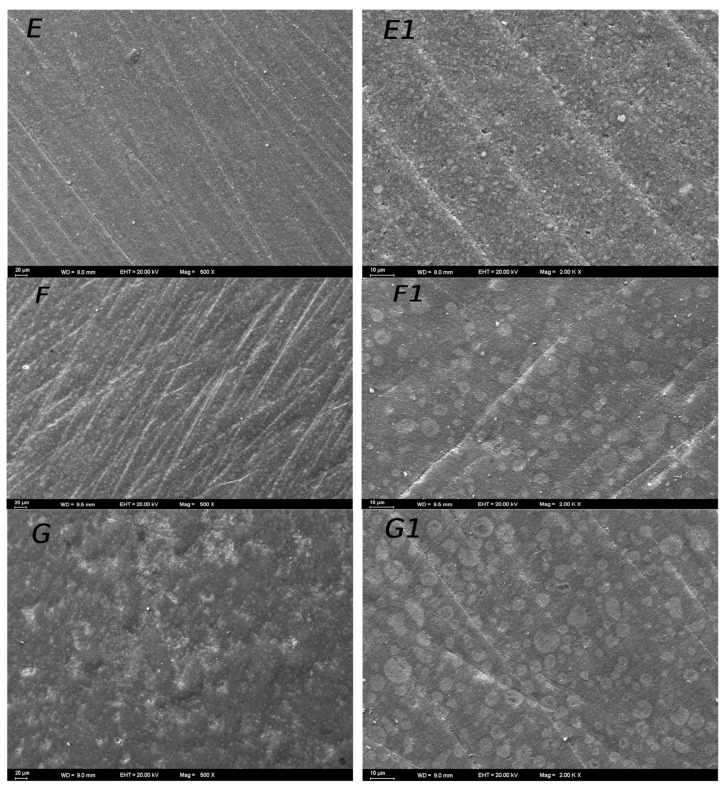
SEM images of the twist-polished samples. (**E**) Charisma Bulk Flow One 500×, (**E1**) Charisma Bulk Flow One 2000×, (**F**) Omnichroma Fill Bulk 500×, (**F1**) Omnichroma Fill Bulk 2000×, (**G**) Estelite Bulk Fill Flow 500×, and (**G1**) Estelite Bulk Fill Flow 2000×.

Distinct surface topographies were observed for various combinations of restorative materials. The SEM images of the materials are given in Figure 5 and Figure 6. Regarding the SEM images, the SEM observations confirm that the Mylar strip yielded smoother surfaces (Figure 5A,A1). The analysis of the polished composite surfaces showed that using discs for finishing resulted in scratches. These scratches probably stemmed from the disc edges during usage (Figure 5B,B1,C,C1,D,D1). However, employing twist polishing (Figure 6E,E1,F,F1,G,G1) led to a more irregular and rough surface finish compared to disc polishing. The profilometer data and SEM images generally corresponded well in the study. In the control group, which had no surface preparation, a smooth composite surface with no deformation was observed. In general, as in the Ra results, the twist-polished specimens showed rougher surfaces than the disc-polished specimens. Some parts of the specimens showed minor surface imperfections, but they also appeared to have smooth polished surfaces. Under 2000× magnification, the samples did not show very deep defects. In addition, polishing Charisma Bulk Flow One with discs resulted in more homogeneous samples than the other groups. Although there were generally fine scratches on the disc-polished samples under 500× magnification, deep scratches that could disrupt the structure of the resin composite were not very common when viewed in more detail under 2000× magnification.

## 4. Discussion

Numerous finishing and polishing systems for composite restorations are accessible and documented in the literature [26,27,28]. In the literature review on the subject, no study was found comparing the optical properties of new generation single color shade bulk-fill resin composites OB and CB polished with discs and diamond-incorporated novel two-step polishing systems. 

Since our research results showed that different polishing systems have an effect on the surface roughness and color stability of bulk-fill resin composites, H_A_1 and H_A_2 are accepted. H_A_3 is also accepted, since there is a statistical difference in the surface roughness of single-shade and multi-shade bulk-fill resin composites. H_A_4, which states that single-shade and multi-shade bulk-fill resin composites differ from each other in terms of color stability, was also accepted. The aging of resin composites begins once they are applied in the patient’s mouth. To more precisely mimic this clinical situation, all tested specimens were subjected to an aging process. It was reported that 10,000 cycles are equivalent to one year of clinical function [29]. Dentists generally arrange dental check-ups for patients twice annually. Elmarsafy et al. simulated six months of clinical function by aging their specimens for 5000 cycles [20]. Similarly, in our study, the samples were aged for 5000 cycles to assess the composites after six months of intraoral use. The optical and mechanical properties of resin composites may change when exposed to oral environmental conditions. Color change in resin composite restorations can also be highly exogenous. Various extrinsic and intrinsic factors can influence the quality and color stability of restorations, potentially leading to staining [14,15]. In accordance with prior research, beverages such as coffee, tea, and wine have been shown to induce staining on resin composites [5,16]. To simulate the staining process, the samples were immersed in the staining solution for six days, simulating six months of consumption of the chosen beverage [16]. In the study conducted by Hasanain, the samples were kept in coffee and tea solutions for 6 days. Similar to Ertas et al. and Hasanain, in the present study, the samples were kept in a coffee solution for 6 days [16,30]. Compared to conventional composites, the proportion of the organic matrix is increased while the amount of filler is decreased in flowable composites. This can lead to higher water absorption, making them more susceptible to discoloration. Consequently, in our study, the OB group, which used a flowable resin composite, exhibited more coloration among the other tested flowable resin composites compared to the nanohybrid resin composite Estelite Sigma Quick. Ipek and Bilge examined the color stability of various resin composites after immersion in common beverages and found that the flowable composite group, which used Omnichroma Bulk Flow, experienced a greater color change. This result is consistent with our findings [6]. Furthermore, despite the large particle filler size of OB, it may have shown lower surface roughness values when polished with discs, due to its uniform spherical structure. Re-polishing may be a good option to restore the aesthetics of composite restorations that are not subjected to too much staining. Given this fact, the concluding H_A_5 of this study, that re-polishing improves the color of coffee-stained resin composites, is confirmed. Re-polishing coffee-stained EB and OB surfaces with disc and twist systems, and CB surfaces with discs only, has allowed these composites to regain color stability within a clinically acceptable range. However, although re-polishing with the twist technique improved the color of the coffee-stained Charisma Bulk Flow One, the values obtained were higher than clinically acceptable level. This may limit the use of twist polishing systems in highly aesthetic clinical situations. In some studies, resin composites polymerized under a Mylar strip exhibited the lowest surface roughness values [31,32,33]. Estelite Sigma Quick showed the smoothest surface when examined under a mylar strip in a recent study [34]. Our control group results are consistent with previous studies and exhibited the lowest surface roughness values before and after aging. When the disc groups were examined, the Ra values were at clinically acceptable threshold values both before and after aging. There was no difference between the OB and CB groups, while the Ra measurement of the EB group was higher than these two groups. When the twist groups were analyzed, the Ra value of all the three tested groups exhibited Ra values above the threshold value before and after aging. The polishability of resin composites is usually related to many factors related to their structural properties. However, it has been shown that Ra is not only dependent on the type of restorative material; additionally, the polishing systems used also have a significant effect on Ra [35]. The content of the polishing material and the hardness and grain size of the abrasive discs have an impact on surface roughness. Removing part of the surface during the finishing and polishing processes can cause microcracks and irregularities [36]. The polishers are tailored for each stage of the process, ensuring optimal results, even with extremely hard composites. Key features include a single instrument suitable for all dental surfaces and the preservation of surface structure during polishing. According to the literature reviews, flexible discs containing aluminum oxide have been shown to produce the smoothest surfaces in resin composite restorations [37,38]. Super-Snap discs coated with aluminum oxide particles and Diacomp plus Twist containing diamond grains were used in this study. Super-Snap discs are used in a multi-step polishing process to achieve a high-gloss finish on composite restorations. These abrasive discs, featuring aluminum oxide grains, possess a Mohs hardness rating of 9, making them potentially ideal for uniformly removing filler particles and the resin matrix from the tested materials during polishing [37,39]. Tepe et al. reported Ra values below 0.3 μm after polishing with Super-Snap discs [40]. Similarly, the specimens polished with the Super-Snap discs gave smoother results throughout this study. In contrast, Diacomp plus Twist is a diamond-incorporated two-step polishing system with a Mohs hardness of 10, and this system yielded rougher surfaces than Super-Snap disc groups after polishing. The hard diamond abrasives of the twist may cause deeper scratches on the composite surfaces than discs. In parallel with Mukhija et al.’s study, it can be said that polishing systems coated with diamond particles induce more profound scratches on dimethacrylate-based resin composite surfaces during polishing, leading to increased roughness in the examined materials [39].

When the SEM findings of the study were analyzed, the disc-polished specimens showed smoother surfaces than the twist-polished specimens in all resin composite groups. Overall, the Ra profilometric findings showed a strong correlation with the SEM images. The CIEDE2000 (ΔE_00_) formula was employed to assess the color of the resin composite [13,41]. The aim of the color difference formula is to offer a quantitative depiction (ΔE) of the perceived color difference between a pair of colored samples under specific experimental conditions. Perceptibility hinges on discerning the color variance between the tooth and its restoration, whereas acceptability centers on the visual harmony of the tooth’s color. The thresholds for perceptibility and acceptability stand at 0.8 and 1.8, respectively [25].

In the present study, when ΔE_00_ values were examined for color measurements before and after aging, the control group differed from the other groups statistically. Except for the OB group, all the other disc and twist groups exhibited clinically acceptable ΔE_00_ values, which are below the detectable threshold value. When the ΔE_00_ values of the disc and twist groups were examined after being kept in the coffee solution, all groups exceeded the visually detectable threshold value and became clinically unacceptable. Water absorption can cause microcracks at the interface between the resin composite and the matrix, resulting in stain penetration and discoloration in the voids [42]. In intraoral conditions, resin composites, due to the colorants in the coffee, can cause absorption to the resin surface and consequently undesirable discoloration [43].

Re-polishing is a minimally invasive method that can remove external discoloration on restoration surfaces. This method can prevent the replacement of resin composites that have been exposed to external discoloration [44,45]. In this study, all of the specimens re-polished with discs were successful and reached a clinically acceptable level except the CB specimens polished with the twist technique. Although there were some limitations, this in vitro study examined the impact of polishing materials on single-shade and multi-shade bulk-fill resin composites.

However, further research using different polishing materials is necessary, and both in vivo and additional in vitro studies should be conducted, as surface irregularities in the oral environment can lead to color changes. Furthermore, in this study, the SEM findings of polished specimens were examined before the aging process. Future research may perform SEM analyses of both polished and unpolished samples before and after aging. Another limitation is that only coffee was used to investigate the color change in the study. The effect of resin composites using different solutions may be investigated in further studies. Lastly, there is a scarcity of studies on single-shade bulk-fill composites in the literature, indicating the need for further research on these resin composites.

## 5. Conclusions

Within the confines of this study’s limitations, the ensuing outcomes were derived:

(1) The polishing method, aging, and staining with coffee significantly affect the surface roughness and color stability of resin composites.
(2) The tested specimens polished with the multi-stage Super-Snap discs produce a smoother surface on all bulk-fill resin composites than the specimens polished with the two-stage Diacomp plus Twist (*p* ≤ 0.05).
(3) Polishing helps to change the surface quality of stained restorations, yet it might not be adequate to entirely eliminate stains and restore the coloration to a clinically acceptable level.
(4) The diamond-incorporated two-step twist polishing system, with a Mohs hardness of 10, may be preferred for re-polishing coffee-stained methacrylate-based resin composites to return them to a clinically acceptable level.
(5) Further research is needed with various polishing materials, along with more in vivo and additional in vitro studies, to address the issue of surface irregularities causing color changes in the oral environment. Additionally, the impact of factors like mechanical stresses, pH variations, and enzymatic activity in the oral environment should be thoroughly investigated.

## Figures and Tables

**Table 1:** Resin composites tested in the study.

Material	Manufacturer	Filler Type	Monomer	Shades	Seri No	Fıller Load	Particle Size
Estelite Sigma Quick (Control)	Tokuyama (Japan)	Silica-zirconia fillers, silica-titania fillers	BisGMA, TEGDMA	A1	E3232	82% wt (71% vol)	0.2 μm
Estelite Bulk Fill Flow (EB)	Tokuyama (Japan)	Supranano-spherical filler	BisGMA, TEGDMA Bis-MPEPP	A1	41912	70% wt (56% vol)	0.2 μm
Charisma Bulk Flow One (CB)	Kulzer (Germany)	Ba-AI-F silicate glass, YbF3 and SiO2	UDMA, EBADMA	Universal	NO10026	65% wt, (41%vol)	0.02–5 μm
Omnichroma Flow Bulk (OB)	Tokuyama (Japan)	Supranano- spherical filler	UDMA/1,9-Nonanediol Dimethacrylate	Universal	011E03	69%wt (55 vol%)	0.2–0.4 μm

**Table 2:** Mean values and SDs of groups for surface roughness (R_a_).

Material	Polishing System	Before Aging	After Aging	*p*-Value
EB	Disc	0.23 ± 0.05 ^b^	0.28 ± 0.04 ^b^	<0.001 *
	Twist	0.42 ± 0.05 ^A^	0.46 ± 0.05 ^A^	
CB	Disc	0.14 ± 0.05 ^a^	0.19 ± 0.05 ^a^	<0.001 *
	Twist	0.27 ± 0.08 ^B^	0.37 ± 0.06 ^A^	
OB	Disc	0.14 ± 0.05 ^a^	0.23 ± 0.05 ^b^	<0.001 *
	Twist	0.35 ± 0.05 ^A^	0.41 ± 0.08 ^A^	

* Significant at *p* ≤ 0.05. Mean ± standard deviation values for differences between measurement times are presented in the table (*p* ≤ 0.05). Different letters indicate the statistically significant differences within the same column (Lowercase letters mean ‘’disc groups’’, uppercase letters mean ‘’twist groups’’).

**Table 3:** Average ΔE_00_ values with standard deviation (SD) of aged, stained, and re-polished materials using two different polishing systems.

Material	Polishing System	Aged (SD)	*p*-Value	Stained (SD)	*p*-Value	Re-Polished (SD)	*p*-Value
EB	Disc	1.83 ± 0.42 ^a^	0.515	2.90 ± 0.45 ^a^	0.906	1.60 ± 1.27 ^a^	0.293
	Twist	1.78 ± 0.39 ^A^		2.92 ± 0.66 ^A^		1.42 ± 0.72 ^A^	
CB	Disc	1.56 ± 0.32 ^a^	0.654	3.33 ± 0.8 ^a^	0.128	1.40 ± 0.58 ^a^	0.013 *
	Twist	1.62 ± 0.45 ^A^		2.87 ± 0.87 ^A^		2.19 ± 1.02 ^A^	
OB	Disc	3.06 ± 0.54 ^b^	0.930	3.75 ± 0.70 ^b^	0.445	1.42 ± 0.76 ^a^	0.293
	Twist	3.05 ± 0.41 ^B^		3.91 ± 0.57 ^B^		1.77 ± 1.05 ^A^	

* Significant at *p* ≤ 0.05. Mean ± standard deviation (SD) values for differences between measurement times are presented in the table (*p* ≤ 0.05). Different letters indicate the statistically significant differences within the same column (Lowercase letters mean ‘’disc groups’’, uppercase letters mean ‘’twist groups’’).

## Data Availability

Co-author Nuran Ulusoy has published the article below in *Pathogens* in 2019. Karadağlıoğlu, Özgü Ilkcan, Nuran Ulusoy, Kemal Hüsnü Can Baser, Azmi Hanoğlu, and Irem¸Şık. “Antibacterial activities of herbal toothpastes combined with essential oils against ˙Streptococcus mutans”. Pathogens 8, no. 1 (2019): 20. Co-author Nuran Ulusoy has published the article below in *Materials*, 2023. Atasayar E., Ulusoy N. “The Effect of Blocker Application on Color Matching of Different Colored Composite Resin Systems”.
